# Laser-assisted blastocyst dissection and subsequent cultivation of embryonic stem cells in a serum/cell free culture system: applications and preliminary results in a murine model

**DOI:** 10.1186/1479-5876-4-20

**Published:** 2006-05-08

**Authors:** Noriko Tanaka, Takumi Takeuchi, Queenie V Neri, Eric Scott Sills, Gianpiero D Palermo

**Affiliations:** 1 Center for Reproductive Medicine and Infertility, Weill Medical College of Cornell University, New York, NY 10021, USA; 2 Department of Obstetrics, Gynecology and Reproductive Research, Murphy Medical Center, Murphy, NC, USA

## Abstract

**Background:**

To evaluate embryonic stem cell (ESC) harvesting methods with an emphasis on derivation of ESC lines without feeder cells or sera. Using a murine model, laser-assisted blastocyst dissection was performed and compared to conventional immunosurgery to assess a novel laser application for inner cell mass (ICM) isolation.

**Methods:**

Intact blastocysts or isolated ICMs generated in a standard mouse strain were plated in medium with or without serum to compare ESC harvesting efficiency. ESC derivation was also undertaken in a feeder cell-free culture system.

**Results:**

Although ICM growth and dissociation was comparable irrespective of the media components, an enhanced ESC harvest was observed in our serum-free medium (*p *< 0.01). ESC harvest rate was not affected by ICM isolation technique but was attenuated in the feeder cell-free group.

**Conclusion:**

Achieving successful techniques for human ESC research is fundamentally dependent on preliminary work using experimental animals. In this study, all experimentally developed ESC lines manifested similar features to ESCs obtained from intact blastocysts in standard culture. Cell/sera free murine ESC harvest and propagation are feasible procedures for an embryology laboratory and await refinements for translation to human medical research.

## Introduction

Embryonic stem cells (ESC) may be produced from the inner cell mass (ICM) of intact blastocysts [1,2], by immunosurgery [3,4], by other methods to isolate pluripotent cells constituting the ICM [5] or single blastomeres [6,7]. Such techniques to derive ESC are easily reproduced in a murine model, the mouse 129 strain being perhaps the most commonly used experimental animal for this purpose [1,5,8,9]. Indeed, this particular mouse model demonstrates a number of desirable ICM features that make it well-suited for laboratory use including rapid cellular growth, relatively large size, and a high content and persistence of stem cells [10,11].

To be sure, the stem cells are not the only murine contribution to ESC experimentation, as mouse embryonic fibroblasts (MEF) also play a central supporting role in the laboratory as feeder cells for ESCs [12,13]. However, a reliance on cell/serum based mouse systems has underscored some important limitations including possible xenogenic or allogenic contamination [4]. Common immunosurgical methods for ICM isolation incorporate the use of allogenic antibodies and complement [3] which may also introduce unwanted epitopes rendering some ESC derivatives unsuitable for further applications.

Here we describe production of ESCs from blastocysts obtained from a standard mouse strain in the absence of feeder cells or sera, with an emphasis on a laser-based ICM isolation modality. Additionally, the cells so harvested met the morphological criteria and growth patterns expected of ESCs as shown by specific molecular markers and by gene expression analysis. Finally, to confirm the ability of our ESC lines to provide the full range of differentiated tissue types, the stem cells were induced to develop into each of the three fetal germ layers.

## Materials and methods

### Specimens

B_6_D_2_-F_1 _(C57BL/6J ♀ × DBA/2J ♂) mice were obtained from Jackson Laboratory (Bar Harbor, ME, USA) and Charles River Laboratories (Wilmington, MA, USA) and housed at the Research Animal Resource Center of Weill Medical College of Cornell University, in a temperature- and light-controlled room on a 14 h light:10 h dark photoperiod with food and water *ad libitum*.

### Embryo collection, culture and media

Females (7–9 weeks) were superovulated with 10 IU of pregnant mare's serum gonadotrophin (Sigma, St. Louis, MO) then 10 IU of hCG (Sigma) 48 h apart. They were next placed with males of the same strain and mating was confirmed by presence of a vaginal plug the following morning. Zygotes collected approximately 20 hours after hCG were cultured in KSOM^AA ^medium (Specialty Media, Phillipsburg, NJ) at 37°C with 6% CO_2 _in humidified air. Our ESC medium consisted of Dubelcco's Modified Eagle's Medium (DMEM, Invitrogen, Carlsbad, CA, USA), 10% fetal bovine serum (FBS, HyClone, Logan, UT, USA), and 10% newborn calf serum (NCS, HyClone) supplemented with mouse leukemia inhibitory factor (LIF, 2000IU/ml, Chemicon International, Temecula, CA), 0.1 mM nonessential amino acids (NEAA, Specialty Media), 0.1 mM β-mercaptoethanol (β-ME, Sigma), and 50 U/ml penicillin/50 μg/ml streptomycin (Sigma). This investigation also utilized Knockout™ DMEM (Ko-M, Invitrogen) with 15% Knockout ™ serum replacement (Ko-S, Invitrogen) containing LIF, NEAA, β-ME, antibiotics, and 4 mM L-glutamine (Sigma) as the supplementary medium.

### ESC harvesting

Feeder layers of mitomycin C-treated MEFs were prepared on either 0.1% gelatin treated 4-well culture dishes (Nalge Nunc International, Rochester, NY) or in 30 μl microdrops under oil in tissue culture dishes. Intact expanded blastocysts (controls) and also zona-free blastocysts and ICMs isolated after immunosurgery or microdissection (see below), were plated onto MEF monolayers and cultured in ESC medium in 6% CO_2 _at 37°C. Blastocysts were observed every 24 h for hatching and attachment of the trophoblast to a single MEF layer, while monitoring ICM size in two dimensions. On day 4 or 5 after plating (D4 or D5), ICM of at least 100 μm were dissociated using a glass pipette, followed by trypsinization in PBS containing 250 U/ml trypsin and 1 mM EDTA (PBS-try-EDTA) to promote cell dispersion. After re-plating in fresh wells coated with feeder cells, ESC colonies developed in 2 or 3 days after which they were trypsinized and propagated by passaging every 2–3 days thereafter. All established cell lines were tested for mycoplasma using MycoAlert ™ Mycoplasma Detection Kit (Cambrex, Rockland, ME, USA).

### ICM isolation

**Figure 1:**
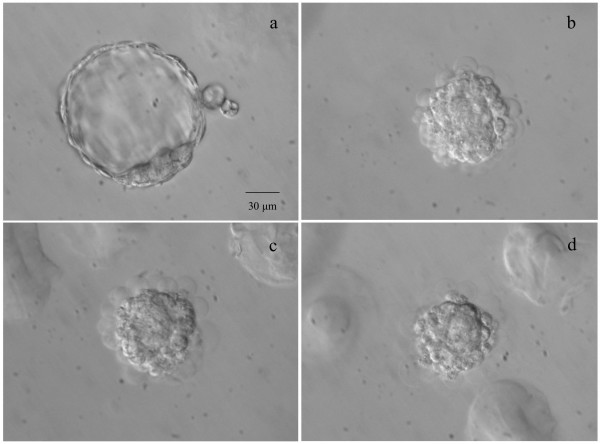
**Immunosurgery**. Zona-free blastocyst (a) coated with rabbit anti-mouse antibodies. After addition of guinea pig complement, the outcome of unselective toxicity can be seen at *t *= 5 min (b), 15 min (c) and 30 min (d). Scale = 30 μm.

**Figure 2:**
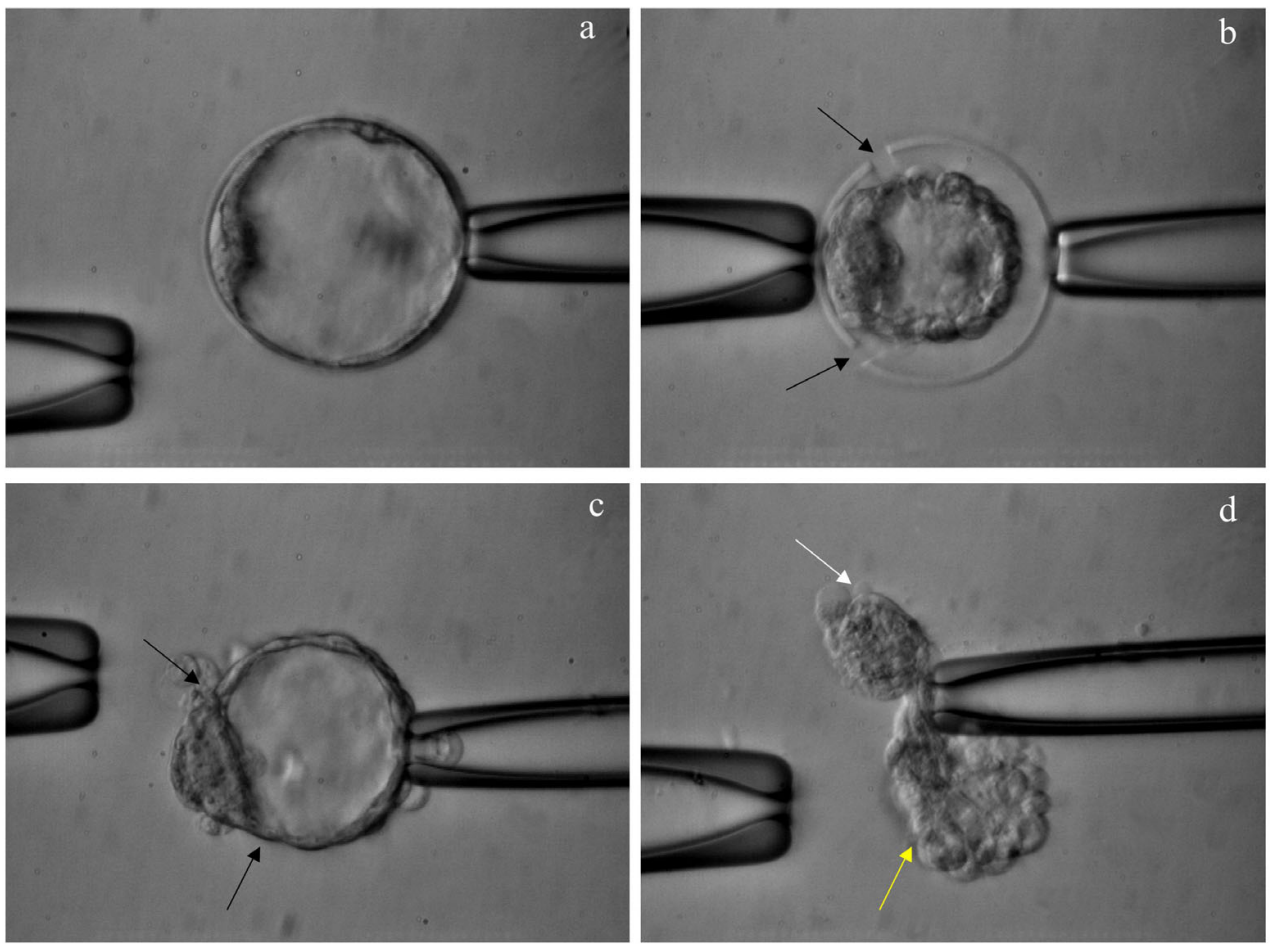
**Laser dissection**. Blastocyst secured by two holding pipettes with inner cell mass (ICM) being positioned at 9 o'clock before (a) and after (b) being sectioned by laser with (b) and without (c) zona pellucida. Arrows (b, c) indicate the resected area by laser energy. The smaller blastocyst fragment (white arrow) contains the ICM while the larger (yellow arrow) is exclusively trophoblast (d). Scale = 30 μm.

Intact blastocysts were plated onto feeder layers on post fertilization day 4–5. Zona-free blastocysts were obtained from incubation of day 4 blastocysts for 4–5 min in 24 U/ml pronase (Sigma). These were washed x2 and cultured for at least 1 h in medium with serum supplementation. To obtain ICMs, zona-free blastocysts were incubated with heat-inactivated rabbit anti-mouse serum (Sigma) diluted with DMEM (Invitrogen) in 1:25 for 15 min then exposed to guinea pig complement (Cedarlane laboratories, Hornby, Ontario, Canada) at 1:10 dilution for 30 min in 6% CO_2 _in air at 37°C (Fig. 1). The disrupted trophoblast was then removed by aspiration through a hand-pulled 50–60 μm (inner diameter) pipette [3]. Laser-assisted ICM isolation was performed as follows: Blastocysts were secured by two holding pipette with the ICM being positioned at 9 o'clock. Once adequate tension was established, approximately 10 infrared laser pulses (300 mW × 1 ms, ZILOS-tk™, Hamilton Thorne Research, Beverly, MA USA) were fired to split the blastocyst into two unequal portions – the smaller consisting of ICM, the larger consisting exclusively of trophoblast (Fig. 2).

### Feeder cell-free culture and ESC characterization

**Figure 3:**
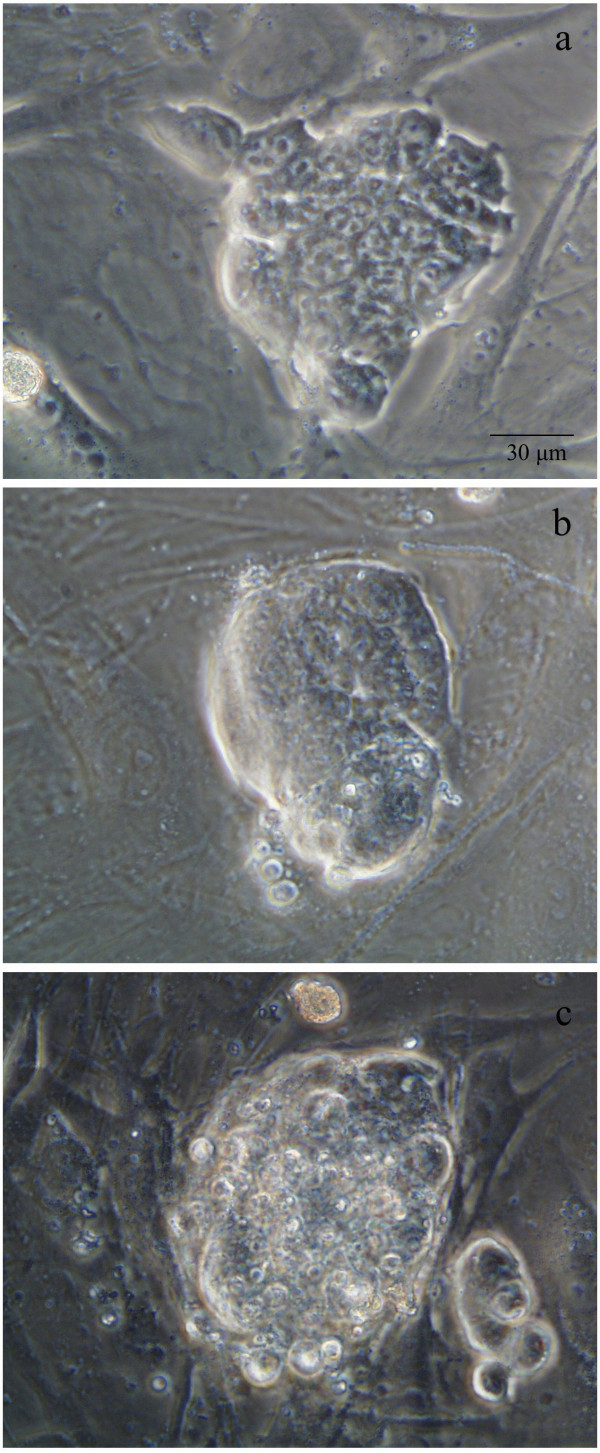
**Grading embryonic stem cell colonies**. Examples of ESC classification into one of the three grades: Grade Good (GG), Average (GA), or Poor (GP). In GG colonies the area occupied by pluripotent cells is >70% of the entire colony (a). Approximately 60% of the colony is occupied by pluripotent cells in GA colonies (b), and a GP colony features only ~20% of pluripotent cells (c). Scale = 30 μm.

The laser dissected ICM components were randomly plated either on MEF feeder layer or directly on gelatin-coated dishes, and cultured in Ko-M with 15% of Ko-S (serum free) containing LIF, NEAA, β-ME, antibiotics, and L-glutamine as described above. Intact blastocysts on MEF were used as controls. ICM size was measured daily, ICM dissociation and ESC propagation was carried out as described earlier. Pluripotency was evaluated according to cell size, nucleus/cytoplasm ratio, and nucleolar patterns. ESC colonies were graded according to number, density, and quality. Colony character was judged under an inverted microscope equipped with phase-contrast optics and stratified as follows: good (GG), average (GA), or poor (GP) according to colony composition of >70%, 40–70%, and <40% pluripotent cells, respectively (Fig. 3). Typically, pluripotent cells are large and have a high nucleus/cytoplasm ratio with one or more distinct nucleoli. DAPI banding was performed on metaphase chromosomes for ploidy assessment.

For this investigation, ESC pluripotency was determined by two positive markers: alkaline phosphatase (AP) activity [8,14] and Oct-4 [15-18]. TROMA-1 monoclonal antibody (Ab) directed against cytokeratin-like filaments present in trophectoderm and endodermal cells was used as a negative marker [8,19-21]. All markers were tested on control blastocysts. Specimens were fixed with 2% paraformaldehyde (Sigma) and permeabilized with 0.5% Triton X-100 (Sigma). AP activity in fixed cells was detected using an azo-dye technique with a Texas-Red filter under fluorescence. The stain solution contained naphthol AS-MX phosphate (Sigma) and fast red TR salt (Sigma) [22]. To detect expression of Oct-4 and TROMA-1, after incubation at 20°C for 60 min in 0.1% bovine serum albumin (BSA, Sigma) and goat serum, ESCs were exposed to Oct-4 polyclonal Ab (Santa Cruz Biotechnology, Santa Cruz, CA) at 1:100 dilution and to monoclonal TROMA-1 Ab (Developmental Studies hybridoma Bank, Iowa City, IA) at 1:6 dilution. After rinsing unbound Abs with PBS/BSA, specimens were next incubated with Alexa Fluor^® ^488 (Invitrogen) or FITC conjugated secondary Abs (Chemicon). The nuclei were counterstained with 0.125 μg/ml of DAPI (Molecular Probes, Eugene, OR) in antifade solution or Topro-3 (Molecular Probes) at 1:500 dilution. Specimens were examined via phase-contrast, fluorescent, or laser confocal microscopy.

### ESC gene expression

Pluripotency confirmation of putative ESCs was by morphological criteria, specific molecular markers, and expression of typical marker genes. *Nanog *(a divergent homeodomain protein that directs propagation of undifferentiated cells) is down-regulated during early de-differentiation and becomes silent in completely differentiated cells [23-25] while transthyretin (*Ttr*, a protein in visceral yolk sac endoderm *in vivo*) [26-28] is expressed in differentiated endoderm cells [29,30]. RNA was isolated from ESCs by Absolutely RNA^® ^Nanoprep Kit (Stratagene, La Jolla, CA) with MEFs serving as a negative control. RNA was stored at -80°C for qualification analysis. Primers were custom-designed by OligoPerfect Designer software (Invitrogen) for the target sequences of *Nanog *and *Ttr *genes, while *Act-β *and *Gapdh *were used as normalizers. Quantitative real-time PCR (qRT-PCR) was performed using SuperScript™ III Platinum^® ^Two-Step qRT-PCR Kit with SYBR^® ^Green (Invitrogen). Analysis was performed using an ABI Prism 7900 HT (Applied Biosystems, Foster City, CA). The qPCR results were plotted by the Sequence Detection System Analysis Software (Version 2.0, Applied Biosystems). Gene expression was reported as ratio data, calculated from the cycle threshold against *Act-β *considered at 100% expression [31].

### Embryoid body formation

ESCs (*n *~10^7^) were subsequently treated with trypsin at 37°C in 5% CO_2 _for 5–7 min, resulting in detachment/release of intact ESC colonies from underlying cells. Cell aggregates of approximately 50–60 ESCs were placed in 20 μl hanging droplets (DMEM, 20% FBS, 2 mM L-glutamine, 0.1 mM NEAA, antibiotics, and 1 mM β-ME) on a 100 mm^2 ^non-tissue culture lid flipped over a dish containing 10 ml PBS. ESCs were cultured for two days to allow aggregation into spheroid embryoid bodies (EBs). On the third day EBs were transferred to a 60 mm bacteriological petri dish in 5 ml of DMEM + 20% FCS, then after two days placed in gelatin coated dishes for five days [32]. Differentiation into myocardiocytes was determined by presence of pulsatile contractility at day 8–9 of culture, at which point the EBs were processed for histological sections and transmission electron microscopy.

### Determination of cellular differentiation

Induction of testicular teratoma formation was used to assess differentiation capacity of experimental cell lines. Approximately 1 to 4 × 10^6 ^undifferentiated cells were injected into the testes of six- to eight- week-old severe combined immunodeficiency (SCID) mice (C.B-*Igh*-1^b^/IcrTac-*Prkdc*^*scid*^, Taconic, Germantown, NY, USA). Three to five weeks later tumors were fixed in 4% paraformaldehyde, embedded in paraffin, and examined histologically after hematoxylin and eosin staining.

### Data analysis

A χ^2 ^test was utilized for comparisons involving blastocyst culture, attachment, ESC derivation, and gene expression analysis. A two-tailed test was used to assess significance, considered at 5% probability. Statistical comparisons were reported in text and tables only when significance was reached. Data tables show numbers within rows with different superscripts as significantly different. All statistical computations were performed with StatView 512+ (BrainPower Inc., Calabasas, CA, USA).

## Results

### ESC harvest

**Figure 4:**
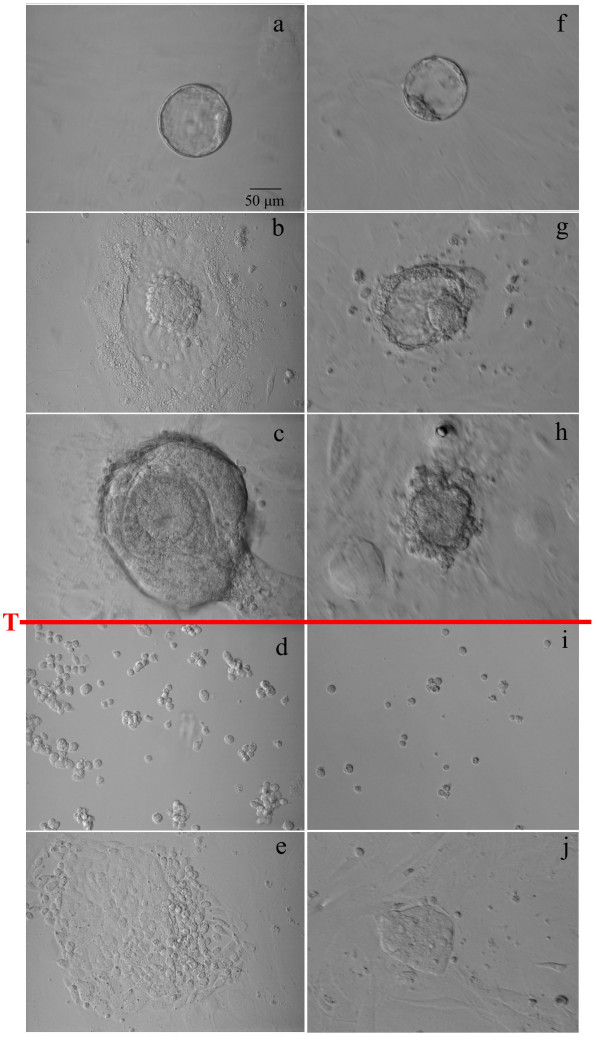
**ICM growth pattern as a function of media**. Inner cell mass (ICM) growth patterns of intact blastocysts in standard DMEM medium (a-e) and in knockout culture system (f-j). Intact blastocysts plated on feeder cells (a, f). ICM attachment and trophoblast outgrowth (b, g). Multilayer differentiation of ICM on day 5 (D5) after plating, note endodermal "rind" (c). Immediately after first dissociation (d, i) blastocysts cultured in standard DMEM failed to produce ESC colonies (e) and generated epithelioid cells. In the knockout system, ICM growth showed negligible endodermal contamination (h) and after first dissociation with trypsin (red T bar) (i) generated ESC colonies (j). Scale = 50 μm.

In a series of experiments, 34 mated mice produced a total of 613 zygotes, 566 (92.3%) of which developed to blastocyst stage after four days of culture. Five preliminary studies were performed in which 126 intact blastocysts were plated on MEFs and cultured in DMEM supplemented with serum. Two days later, 82 (65.1%) ICMs had hatched and attached to the feeder cell layer. On plating day 5 (D5), 71 ICMs (56.3%) were successfully dissociated and replated, However, no ESC lines were established. Optimal medium for murine ESCs was determined from nine experiments where 126 intact blastocysts were allocated to DMEM + 20% serum (Fig 4a–e) vs. Ko-M + 15% Ko-S (Table 1) (Fig.4f–j). In both media, over 60% of the plated blastocysts attached to the MEF layer and it was possible to isolate between 20 to 30% of the ICMs. While only 1 ESC line (1.6%) was harvested from the standard DMEM system, 11 (17.5%) were obtained from the Ko-M medium (*p *< 0.01). The culture carried out in microdrops yielded comparable hatching and attachment rates, although it facilitated easier monitoring of ICM attachment and assessment of development than the 4-well plate system. Although gelatin treatment was technically difficult for microdrop stabilization and positioning, gelatinization of the area before preparation of the microdrop areas made this easier.

**Table 1:** Intact blastocyst culture on MEF in different media

Embryonic day (d)	Plating day (D)	No. of (%)	DMEM + 10%NCS, 10% FBS	Ko-M + 15% Ko-S
d4	D0	Blastocysts (I) plated	63	63
d5–6	D1–2	Hatched blastocysts	53 (84.1)^a^	42 (66.7)^b^
d6–7	D2–3	Attached on MEF	51 (80.9)	42 (66.7)
d8–9	D4–5	ICM isolation	18 (28.6)	14 (22.2)
d14–16	D10–12	ESC primary colonies	1 (1.6)^c^	11 (17.5)^d^

FBS = fetal bovine serum, NCS = newborn calf serum, Ko-M = Knockout™ DMEM, Ko-S = Knockout™ serum replacement

^a,b^χ^2^, 2 × 2, 1 *df*, Effect of ESC medium on hatching rate, *p *< 0.05

^c,d^χ^2^, 2 × 2, 1 *df*, Effect of ESC medium on ESC derivation rate, *p *< 0.05

### ICM isolation and ESC characterization

**Figure 5:**
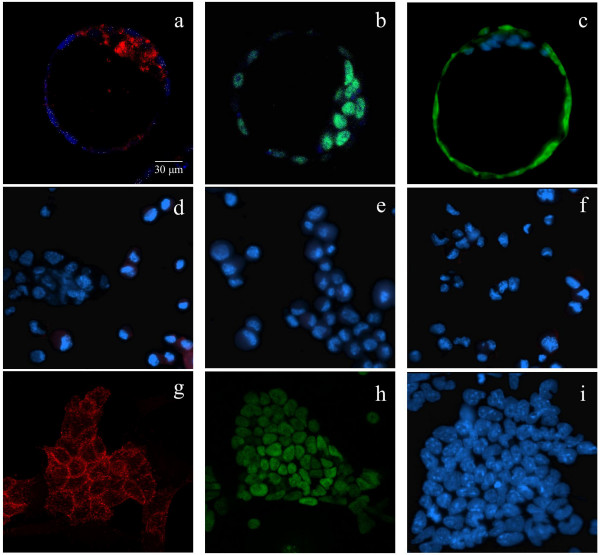
**Localization of pluripotency markers**. Alkaline phosphatase (AP) activity (left), Oct-4 (center), and TROMA-1 (right) expression as measured in expanded and hatched blastocysts (top row, a-c), mouse embryonic fibroblasts (middle row, d-f) and embryonic stem cell colonies (bottom row, g-i) by immunofluorescent analysis. In left column, red signals indicate positive AP activity (a, g). In center, red signals identify positive Oct-4 expression (b, h) while the right column TROMA-1 expression is shown as green signals (c, hatched blastocyst). DAPI was used for nuclear DNA counterstaining as indicated by blue signals. Scale = 30 μm.

ICM attachment ability and consequent ESC colony harvest according to the different embryo manipulation methods vs. control (*n *= 84) are depicted in Table 2. Zona removal enhanced ICM attachment and became significant (*p *< 0.05) when operative isolation of the ICM was carried out. Embryo manipulation including operative isolation of the ICM did not affect harvesting rate nor quality and grading of ESC colonies. For these studies, morphology was a consistent and reliable criterion for evaluating ESC characteristics, the morphological characteristics of stemness correlated closely with cell markers and gene expression assays. A proportion of GG colonies were not influenced by culture systems or ICM isolation method. Colonies graded as GG had four normal male and one female karyotype. All established ESC lines remained euploid after several passages. Colony pluripotency was confirmed by molecular markers. Outcomes using AP and Oct-4 were in agreement (χ^2 ^= 0.105) for determining pluripotency of derived ESC lines. TROMA-1 served as a negative control for endodermal and trophoblastic cell contamination (Fig. 5). Pluripotency marker assessment was concordant in all ESC colonies (Table 3). Multimarker assay confirmed the ability of the morphological criteria and grading system to identify pluripotent cells. We processed 10,000 to 30,000 cells to generate 0.3 to 0.9 μg of total RNA that displayed two ribosomal subunits. Reverse transcription and qRT-PCR were successful in all cases. As expected, *Nanog *was expressed in 74.6% of ESCs, but not in any MEFs. The latter manifested a higher expression (69.6%, *p *= 0.0001) of *Ttr *(denoting differentiation) compared to ESC colonies (40.7%).

**Table 2:** Influence of blastocyst manipulation on ICM attachment and ESC harvesting

	Blastocyst manipulation methods			
	
No. of (%)	IC	ZF	IS	LD
Blastocysts/ICM	25	14	24	21
Attached on MEF	17 (68.0)^a^	12 (85.7)^a,b^	24 (100)^b^	20 (95.2)^b^
First dissociation	6 (24.0)	3 (21.4)	9 (37.5)	9 (42.8)
ESC primary colonies	4 (16.0)	1 (7.1)	3 (12.5)	5 (23.8)

IC = Intact blastocyst, ZF = Zona-Free blastocyst, IS = Immunosurgery, LD = Laser dissection

^a,b^χ^2^, 2 × 4, 3 *df*, Effect of blastocyst manipulation on ICM attachment, (IC vs IS & LD), *p *< 0.05

**Table 3:** ESC marker expression in derived ESC lines

			Stemness phenotype*		
			
ESC line ID (Experiment# _Blastocyst#_)	Blastocyst manipulation methods	Initial grading	AP activity^≸^	Oct-4^≹ ^	TROMA-1^∫^
16_7_	I	Poor	+	+	-
16_34_	I	Average	+	+	-
16_35_	I	Good	+	+	-
17_6_	ZF	Poor	+	+	-
17_13_	IS	Average	+	+	-

* + and – indicate "positive" or "not detectable" activity or protein expression, respectively

^†^AP activity is positive marker for pluripotent cells

^‡^Oct-4, Octamer-binding transcriptional factor 4, is positive marker for pluripotent cells

^∫ ^TROMA-1, trophectodermal monoclonal antibody, which reacts to keratin filaments in trophectodermal and endodermal cells, is assessed as a negative marker for pluripotent cells

### Differentiation of cells

**Figure 6:**
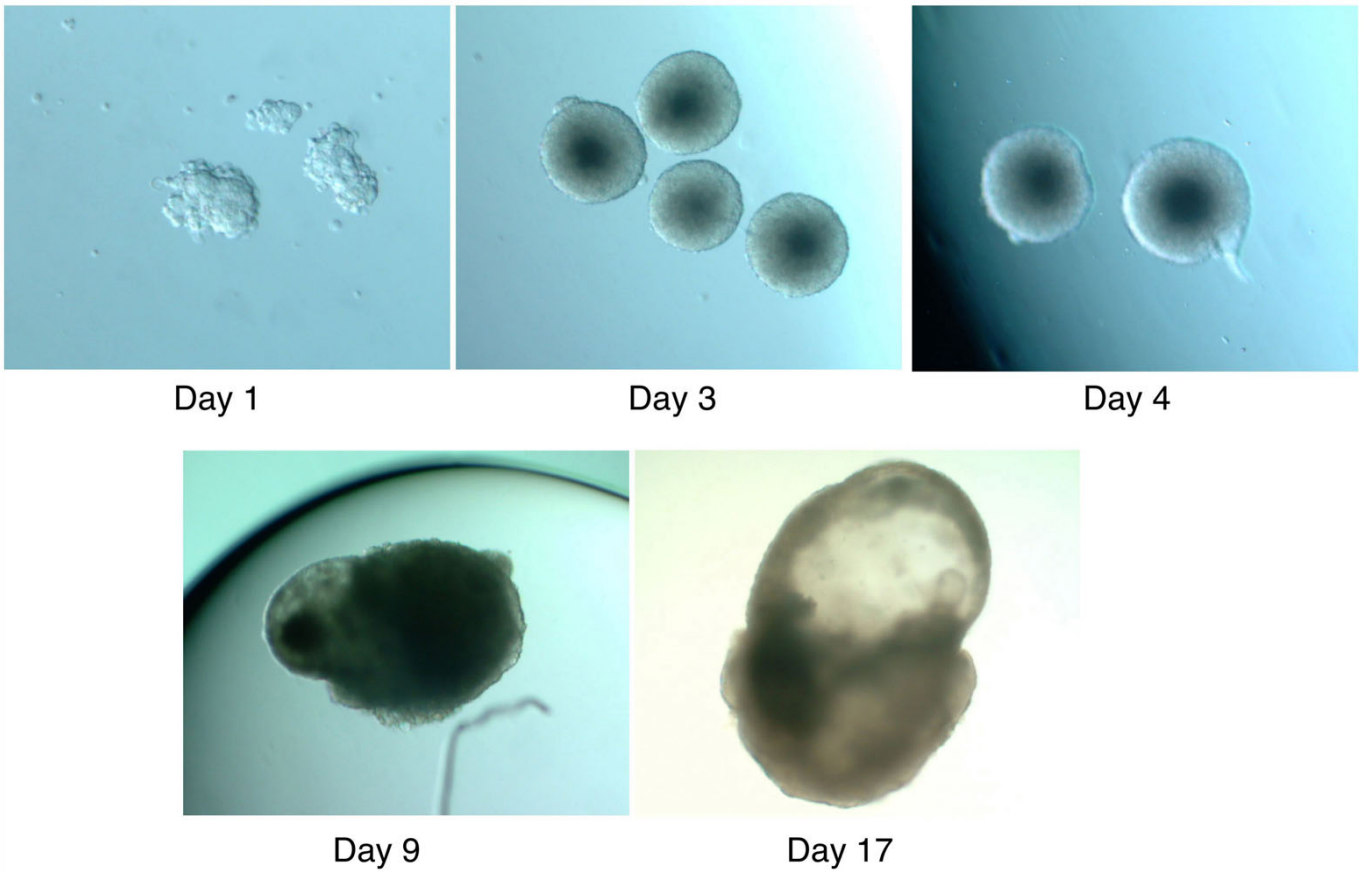
**Coalescence and development of embryoid bodies**. Day 1: Aggregation of individual ESCs to form spherical bodies (EB = embryiod body). Day 3: EBs ranging from 100–250 μm mean diameter. Day 4: Growing EBs of ~350 μm. Day 9: An EB with a cystic structure. Day 17: EB with outgrowth and vesicular cavity.

**Figure 7:**
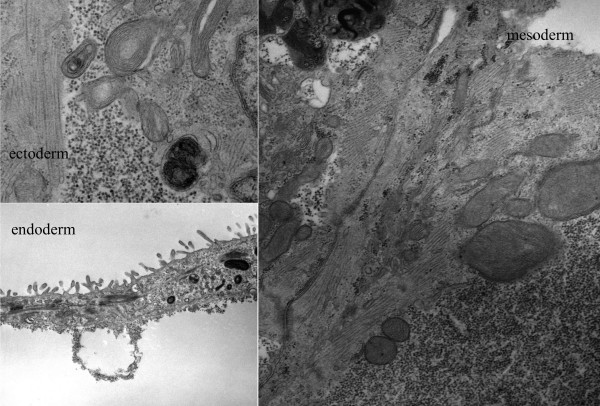
**Ultrastructure of embryoid bodies**. Transmission electron microscopy of embryoid body showing three embryonic germ layers including keratin filaments (ectoderm), cardiomyocyte with contractile components (mesoderm), and microvilli (endoderm).

**Figure 8:**
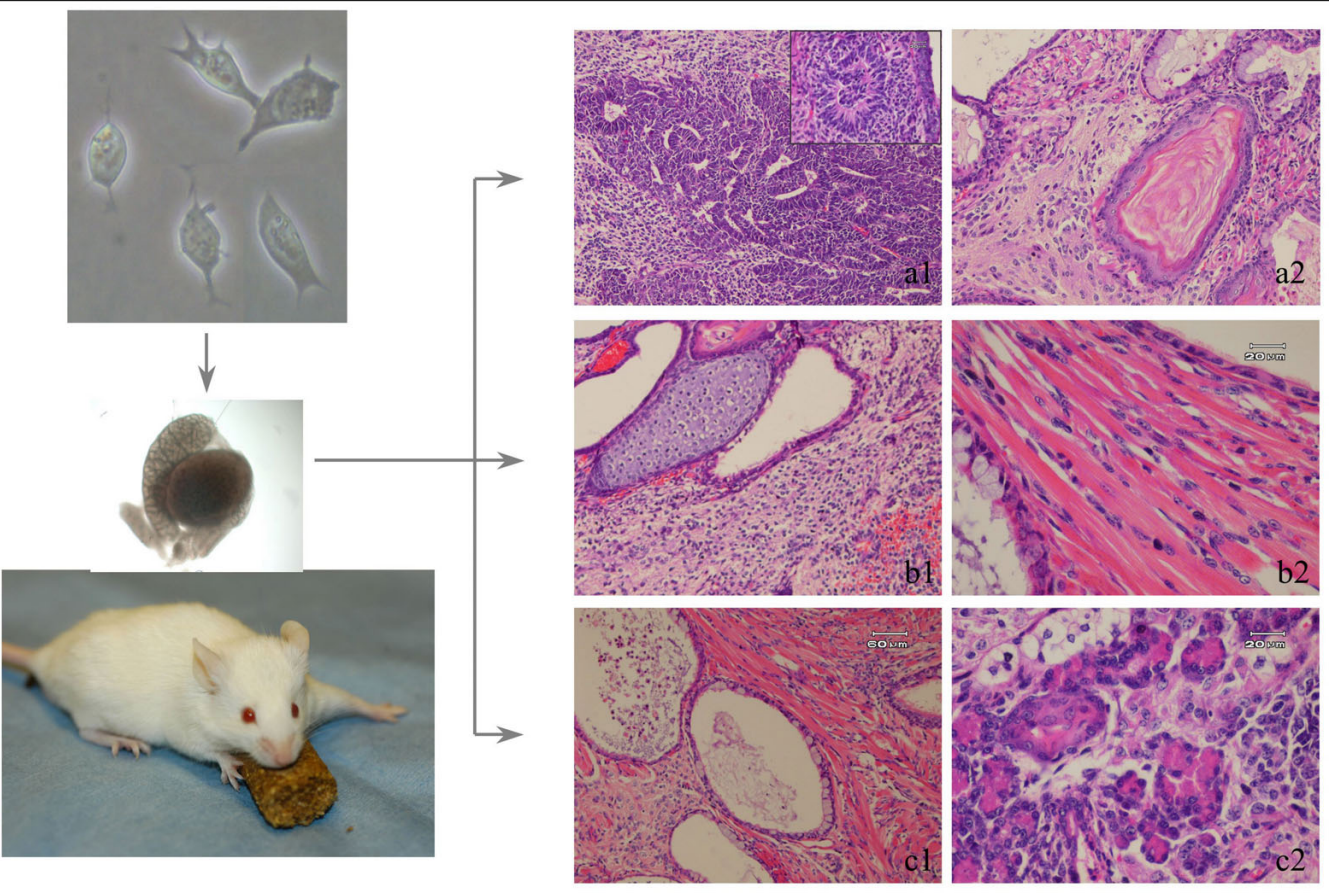
**Histology of teratoma induced by ESC injection**. Teratoma was induced by injection of ESC into SCID mouse testis. Ectodermal differentiation is observed with basophilic staining in a cord-like distribution, typical neural tissue organization (a1), and neuronal rosette (a1 inset). Squamous epithelium shows normal keratin deposition pattern (a2). Mesodermal components include cartilage (b1) and striated muscle (b2). Endodermal differentiation to a gut-like structure lined with mucinous epithelium (c1). Cells organized in acinar structure typical of glands (pancreas or salivary) (c2).

Using morphological determinants and molecular markers, at least 80% of ESC colony cells were undifferentiated. Culture in the absence of feeder cells and LIF permitted formation of tight, round aggregates. After day 2 of culture, a distinctive outer layer of endodermal cell differentiation became apparent, each droplet containing a spherical embryoid body of ~320 μm diameter (Fig. 6). Reaching ~450 μm diameter by day 9 of culture, about 20% EBs displayed foci of pulsatile contraction typical of cardiomyocyte differentiation (Fig. 6). Histology and ultrastructural examination confirmed cell components of primordial embryonic germ layers (Fig. 7). EBs cultured for 16 days displayed widespread epithelial cell differentiation as well as cells lining the lumen of cavity found between the EBs. Both simple and stratified epithelial cells were present. Testes injected with ESCs uniformly developed tumors containing derivatives of all three embryonic germ layers including mucus-producing columnar epithelium, ciliated columnar epithelium, glandular epithelium arranged in acini, endothelium lined vessels within intraluminal neutrophils, striated muscle, bone, cartilage, neural tissue (often forming rosettes), and keratinizing epithelium (Fig. 8).

### Performance of cell- free culture system

Intact blastocysts (*n *= 131) were laser dissected and 65 of these were plated on MEF with the remainder (*n *= 66) on gelatin-coated dishes (Table 4). Laser dissection was successful in all cases. In the absence of MEF, attachment of ICM occurred at a similar rate than control (81.8% vs. 92.3%), and observed growth patterns were also comparable. A total of 6 (9.1%) ESC lines was established on gelatin, while 15 (23.1%) on MEF. ESC harvest efficiency was significantly higher on feeder cells (*p *< 0.05). Cell lines obtained in feeder cell-free conditions maintained pluripotency to the same degree as that observed in controls

**Table 4:** ESC harvesting efficiency with or without feeder cell layers

No of (%)	Feeder	Without feeder
Isolated ICMs	65	66
Attached	60 (92.3)	54 (81.8)
First dissociation	27 (41.5)	21 (31.8)
***ESC colonies***	15 (23.1)^a^	6 (9.1)^b^

^a,b^χ^2^, 2 × 2, 1 *df*, Effect of feeder cell on ESC harvesting, *p *< 0.05

## Discussion

As with any scientific investigation using animal models, it is envisioned that experience gained from murine ESC work will supply answers to challenges still vexing the human ESC field. To be sure, the recent identification of proteins and sialic acid residues on stem cell surfaces [33] resulting from MEF contamination has redoubled the need to develop methods to culture ESCs without feeder cells, and further research is needed to derive ESC lines in more controlled systems [8,13,34]. In our investigation we obtained ESC lines without feeder layers and sera, although the efficiency was lower than desired. These cell lines demonstrated all ESC characteristics with respect to morphology, marker expression, and differentiation capability both *in vivo *and *in vitro*. These results therefore represent a start along the way to establish ESC lines in controlled and xenogenic by-product free culture conditions.

The morphological grading method developed here proved useful to characterize colonies as well as to monitor pluripotent status of the cells, and was helpful to identify early differentiation. All cell lines obtained experimentally demonstrated typical ES morphology via light microscopy, staining strongly for alkaline phosphatase activity and expressing Oct-4 [13]. Molecular markers such as Oct-4 [15,16] and *Nanog *expression [23,24] are critical to confirm stemness because of their role in pluripotency regulation and cellular self-renewal. Assessment of trophoblastic markers such as TROMA-1 [19,20] and *Ttr *[26,27] supported cytometric grading in our derived ESC lines. The development of embryoid bodies was a further indicator that harvested cells retained the ability to develop into the three embryonic disc components. Additionally, the observation that all established cell lines gave rise to teratomas *in vivo *served to confirm their pluripotency.

A recent study [35] described only a 2% rate of ESC derivation using C57BL/6 mice, a model with a similar genetic background compared to our B_6_D_2_-F_1 _(C57BL/6J × DBA/2J) strain. Results from the present study are in general agreement with those of previous reports [36,37] where DMEM + serum was utilized, although substantially lower than the 30% reported from the 129 strain [1]. The higher ESC efficiency reported from the 129 strain may be due to the fact that this is an inbred strain and is characterized by the persistence of stem cells into adulthood [10], or, alternatively, because of absence of a PGC survival factor [38,39]. In our experiments, ESC harvest was improved by substituting a combination of serum free Ko-M + Ko-S media [40], the beneficial effect of which may be ascribed to the relatively low osmolarity and the absence of differentiating factors present in bovine sera [36,41-43].

**Figure 9:**
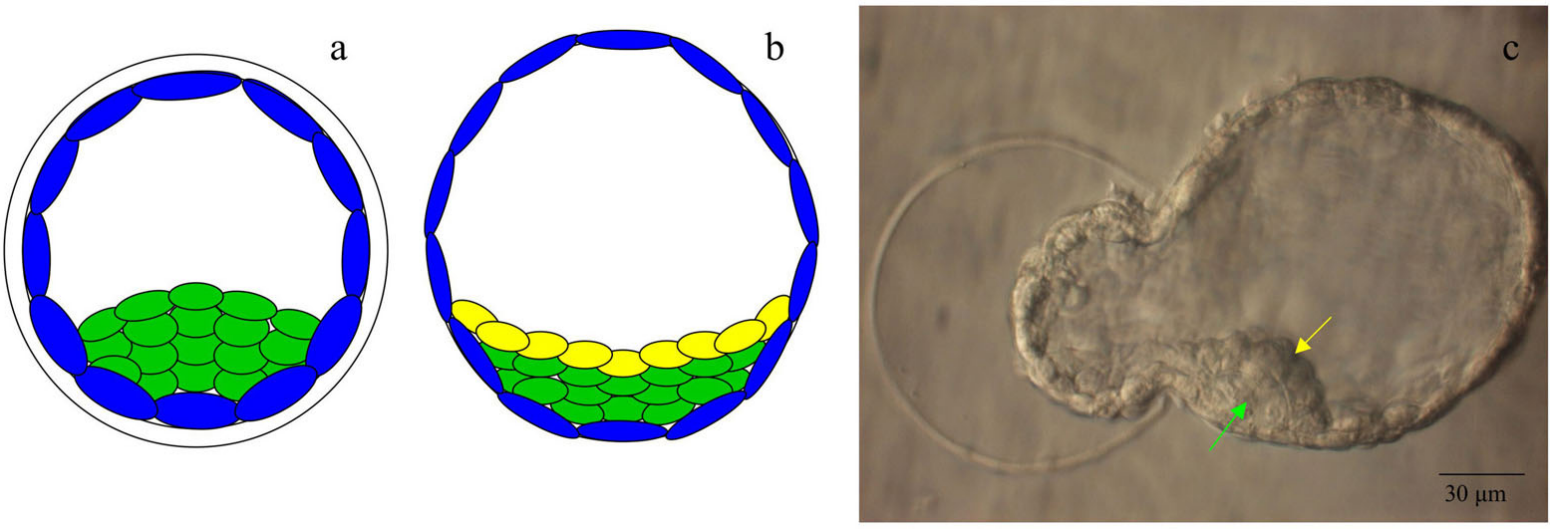
**Organization of primitive endoderm**. Schematic of expanded blastocyst with absence (a) and presence (b) of primitive endoderm (hypoblast) in a day 4 expanded blastocyst and day 6 hatched blastocyst, respectively. In b, ICM remnant is defined as the epiblast (green) and the hypoblast (yellow). Hatching blastocyst (c) with epiblast (green arrow) and hypoblast (yellow arrow). Scale = 30 μm.

**Figure 10:**
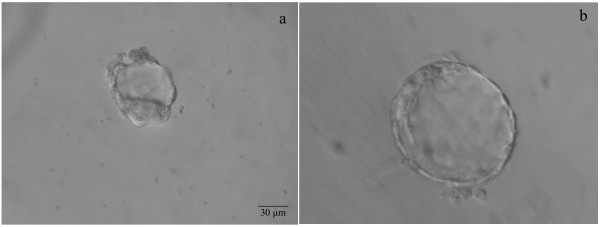
**Vesicle formation post-immunosurgery**. Unilaminar vesicle generated from plated ICM after immunosurgery with obvious endodermal contamination at day 1 (a) and day 2 (b). Scale = 30 μm.

**Figure 11:**
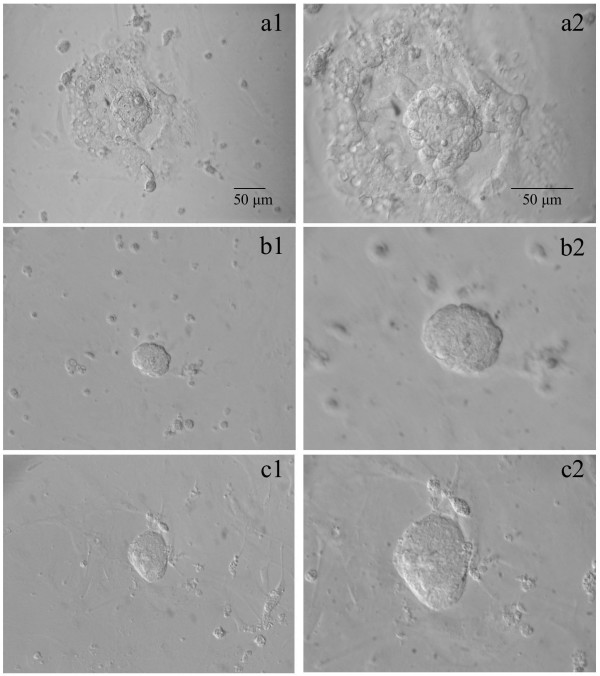
**Observed growth patterns according to ICM isolation technique**. Attachment of ICM and trophectodermal outgrowth with large (b1, b2, after immunosurgery) or minimal (a1, a2, intact blastocyst) endodermal contamination. Such contamination is completely absent following laser dissection (c1, c2). First column shown at 200×, second column shown at 400×. Scale = 50 μm.

Reduced ESC harvesting following immunosurgery might be explained by endodermal contamination (Fig. 9) and/or persistence of trophoblastic cells as proven by the formation of unilaminar vesicles (Fig. 10). Most ICMs isolated by immunosurgery from day 5 blastocysts can regenerate an external layer of endodermal cells once cultured *in vitro *[44], offering further evidence that immunosurgery is not ideal for isolation of uncontaminated epiblasts. As cytotoxic antibodies destroy all blastocyst cell types, a less than perfect timing of exposure to such reagents might result in escape of some endodermal cells [44] allowing them to form the characteristic rind (Fig. 11).

Laser applications have been used in the assisted reproductive technologies for several years, including assisted hatching [45] embryo or polar body biopsy [46,47], sperm immobilization [47], and ICSI [48], all of which have resulted in successful pregnancies. In contrast to immunosurgery where trophoblastic disruption depends on bioactivity of antibodies and complements [49], laser pulses can be precisely delivered to excise the ICM. Perhaps more importantly, ICM isolation via laser energy avoids xenogenic contamination by reagents and requires minimal micromanipulation skills. Although operative isolation of ICM did not translate to a higher proportion of harvested ESCs in the present study (Table 2), identification of other techniques to isolate ESC precursor cells from ICM may reduce embryo wastage [7]. Among various methods tested here, laser dissection seemed to optimize ESC harvest. This observation is in general agreement with previous work [5,50-59] where specific ICM progenitors of stem cells were identified by disaggregation of the epiblast under varied culture conditions. In our studies, this approach yielded a higher ESC harvest rate than that following intact blastocyst plating (52 vs. 23%, respectively). It is anticipated that additional research will further optimize cell-free culture systems [50,53].

## Conclusion

In summary, this research illustrates that while a thorough understanding of culture conditions is essential to develop an effective general strategy for the efficient derivation of mammalian ESC lines, this can be accomplished in a routine embryology laboratory using a conventional mouse strain and laser applications as described here. Using B_6_D_2_-F_1 _mice we established 46 new ESC lines: one via a standard DMEM medium, 16 with a 'knockout' medium, and 29 derived after operative isolation of the ICM – including 6 without feeder cells and serum. While all lines were euploid, exhibited good morphology, and maintained a high level of pluripotency when tested *in vitro*, these results merit continued study to refine establishment of ESC lines from non-permissive mouse strains.

## Competing interests

The author(s) declare that they have no competing interests.

## Authors' contributions

NK coordinated all laboratory work on the project with assistance from QV, ESS, ZR, and GDP. GDP conceptualized the research, reviewed the data, and supervised manuscript preparation.
